# PINK1 signalling in neurodegenerative disease

**DOI:** 10.1042/EBC20210036

**Published:** 2021-12-22

**Authors:** Daniel R. Whiten, Dezerae Cox, Carolyn M. Sue

**Affiliations:** 1 Department of Neurogenetics, Kolling Institute, The University of Sydney and Northern Sydney Local Health District, Sydney NSW 2065, Australia; 2 Department of Chemistry, University of Cambridge, Cambridge CB2 0XY, U.K.

**Keywords:** Mitochondria, Mitophagy, Neurodegneration, Parkinsons disease, PTEN induced putative kinase 1

## Abstract

PTEN-induced kinase 1 (PINK1) impacts cell health and human pathology through diverse pathways. The strict processing of full-length PINK1 on the outer mitochondrial membrane populates a cytoplasmic pool of cleaved PINK1 (cPINK1) that is constitutively degraded. However, despite rapid proteasomal clearance, cPINK1 still appears to exert quality control influence over the neuronal protein homeostasis network, including protein synthesis and degradation machineries. The cytoplasmic concentration and activity of this molecule is therefore a powerful sensor that coordinates aspects of mitochondrial and cellular health. In addition, full-length PINK1 is retained on the mitochondrial membrane following depolarisation, where it is a powerful inducer of multiple mitophagic pathways. This function is executed primarily through the phosphorylation of several ubiquitin ligases, including its most widely studied substrate Parkin. Furthermore, the phosphorylation of both pro- and anti-apoptotic proteins by mitochondrial PINK1 acts as a pro-cellular survival signal when faced with apoptotic stimuli. Through these varied roles PINK1 directly influences functions central to cell dysfunction in neurodegenerative disease.

## Introduction

Despite decades of ongoing work, neurodegenerative diseases are a group of mostly incurable disorders. Many of these disorders, including Alzhiemer's disease (AD), Parkinson's disease (PD) and amyotrophic lateral sclerosis, exhibit shared dysfunctions such as compromised mitochondrial bioenergetics [1] and impaired protein homeostasis (proteostasis) [2]. In these diseases, the dysfunction often stems from distinct genetic mutations, and as such causative pathways appear diverse and complex. However, common impairments and deleterious effects common across distinct pathologies provide insight into human health and help inform therapeutic strategies.

The development and maintenance of functional mitochondria is a critical aspect of cell health. Failure to maintain homeostasis results in the disruption of crucial functions such as the production of ATP through oxidative phosphorylation, intracellular Ca^2+^ signalling and lipid synthesis contribute heavily to a range of human pathologies [3]. Such dysfunctions, particularly in the electron transport chain, can be exacerbated by the enhanced production or limited capture of reactive oxygen species. Excess accumulation of reactive oxygen is a feature common to many neurodegenerative diseases [4]. Therefore, mitochondrial activity is monitored by numerous quality control mechanisms. Chief among these is the selective degradation of compromised mitochondria by the autophagic machinery, a process known as mitophagy [5]. By removing impaired mitochondria, mitophagy can directly alleviate dysfunctional increases in mitochondrial oxidative stress. Mutations in genes (i.e. *PINK1* and *PRKN*) that are intrinsically linked to mitophagy are causal for PD, the second most common neurodegenerative disorder, which is characterised by the progressive loss of dopaminergic neurons in the substantia nigra [6].

*PARK2* was the second gene implicated in PD, following the discovery of α-synuclein as the principal component of PD Lewy bodies [7], and the first known to cause autosomal recessive early-onset PD [8]. Since this time more than 200 *PARK2* mutations have been shown to cause PD. The gene encodes parkin, an E3 ubiquitin ligase that appears to play a crucial role in the turnover of damaged mitochondria. Supporting this notion are mutations in an additional gene, *PARK6* (encoding PTEN-induced kinase 1; PINK1), that also causes autosomal recessive early-onset PD [9] that is often phenotypically similar to cases caused by *PARK2*. Two subsequent landmark studies indicated that both proteins regulate mitochondrial function via the same biochemical pathway [10,11]. It was later reported that both PINK1 and Parkin contribute to PD pathology through a loss-of-function mechanism resulting in impaired mitophagy. Here, we review the multiple roles played by PINK1, including the PINK1/parkin signalling pathway and briefly outline alternative PINK1/Parkin-independent mechanisms of mitophagy that may be leveraged as therapeutics for neurodegenerative disease.

## PINK1 the protein: structure, processing and activation

**Figure 1:**
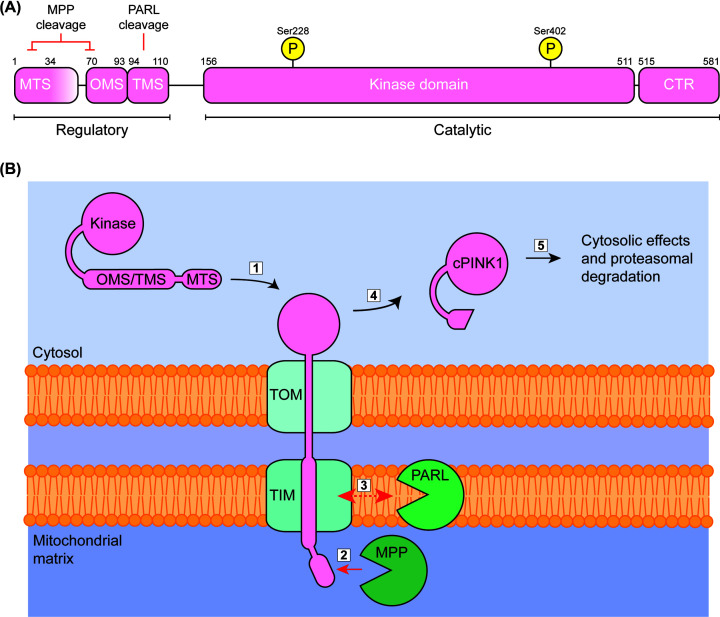
PINK1 domain structure and regular mitochondrial processing (**A**) The PINK1 protein is composed of a regulatory region, consisting of the MTS, OMS and TMS, a catalytic KD and a C-terminal region which aids in substrate recognition. The exact location and length of the MTS is still debated, indicated by the shading. Also indicated are potential MPP cleavage sites, as well as the PARL cleavage site and phosphorylated serines. (**B**) Processing of PINK1 in healthy mitochondria. (1) PINK1 is imported into the mitochondria through the TOM/TIM complex owing to its N-terminal MTS. Cleavage by (2) MPP in the mitochondrial matrix, and (3) PARL on the IMM precede the release of cPINK1 into the cytosol (4). (5) Cytosolic PINK1 can be stabilised by chaperones or ubiquitination by TRAF6 before proteasomal degradation. Abbreviations: cPINK1, cleaved PINK1; PARL, presenilin-associated rhomboid-like protein.

PINK1 is a nuclear-encoded kinase synthesised in the cytosol as a 62.8-kDa polypeptide composed of five regions: (1) an N-terminal domain containing the mitochondrial targeting sequence (MTS); (2) an outer membrane localization signal (OMS) for association with the translocase of the outer membrane (TOM); (3) a transmembrane domain (TMD); (4) a kinase domain (KD) which remains cytoplasmic and (5) a C-terminal region (CTR) which regulates structural arrangement and substrate recognition by the kinase region (Figure 1A) [12,13]. Activation of the Ser/Thr KD requires phosphorylation at Ser^228^ and Ser^402^, and this has been demonstrated to occur via either auto- or trans-phosphorylation [14].

Under physiological conditions the full-length protein (fPINK1) is transported to mitochondria, owing to the N-terminal MTS (Figure 1B). Import across the mitochondrial membrane is facilitated by the translocases of the outer and inner membrane (TOM/TIM), where the first of two sequential cleavage events is mediated by mitochondrial processing peptidases at several possible sites [15]. The second cleavage event takes place at the inner membrane, where presenilin-associated rhomboid-like protein (PARL) protease cleaves between Ala^103^ and Phe^104^ [16] to generate the 52-kDa mature cleaved PINK1 (cPINK1; also known as ΔN PINK1, PINK1-52, sPINK1 and PINK-1-s). cPINK1 is finally retrotranslocated to the cytoplasm where a small population is bound by chaperones [17] and the remainder is efficiently turned over via proteasomal degradation. Degradation of cPINK1 was first attributed to the N-end rule pathway [18], although Liu and colleagues [15] have since provided evidence that challenges this mechanism. Instead, they assert degradation is mediated by the preferential polyubiquitination of K137, a lysine residue exposed by conformational rearrangement in cPINK1, via interaction with the endoplasmic reticulum (ER)-associated degradation pathway [16]. Additional mechanisms have been proposed for fPINK1 degradation, including ubiquitylation by an E3 ubiquitin ligase (Skp1-Cul1-Rbx1) recruited by the F-box protein FBXO7 [17] and direct degradation by the mitochondrial matrix protease Lon [18]. Both fPINK1 and cPINK1 have also been shown to interact with the ATP-independent molecular chaperone BAG6 [19], known for targeting substrates to the proteasome for degradation, which may prove to be a common nexus for basal processing of both PINK1 forms. In any case, the abundance of mature cPINK1 is tightly regulated such that, normally, cytosolic levels are barely detectable via standard biochemical techniques. This balance is cell type-dependent; cytosolic cPINK1 is most readily found in healthy neurons, likely due to the chaperones mentioned above and TRAF6 ubiquitination [19,20]. However, the strict balance and subcellular localisation of fPINK1 and cPINK1 are emerging as an intricate regulatory switch that allows the cell to couple management of proteostasis, energetic demand and mitochondrial integrity. These functions are discussed in more detail below.

## cPINK1 primarily exerts regulatory effects via cytosolic interactions

Constitutive degradation of cPINK1 within the cytosol has rendered efforts to characterise its activity challenging. As a result, many studies have employed overexpression models of cPINK1 in immortalised cell lines. Nonetheless, current experimental evidence paints a picture of a multifaceted signalling molecule whose activity spans the proteostasis network, much of which is independent to the role of fPINK1 in stimulating mitophagy [20] (summarised in Table 1). For instance, cPINK1 is implicated in modulating protein transcription and translation. Direct evidence supporting this function includes cPINK1-induced phosphorylation at Ser^398^ of the translation elongation factor eEF1A1 [21], which results in inhibited protein synthesis under conditions of cellular stress. Phosphoproteomics of cultured cells deficient in PINK1 also revealed an enrichment for nuclear proteins among those whose phosphorylation was altered by the absence of PINK1 [22]. Modified proteins were strongly associated with transcriptional regulation, nuclear–cytoplasmic transport and RNA processing. This is complemented by a recent study [23] in which cPINK1 itself was found to be translocated into the nucleus following mono-ubiquitination under stress conditions. This translocation is yet to be directly linked to the phosphorylation of nuclear proteins by cPINK1, but it certainly provides a tantalising connection for future studies of cPINK1 phosphorylation-mediated transcriptional regulation.

**Table 1:** Potential roles for cPINK1 in mediating cellular proteostasis

Proteostasis activity	Mechanism	Model system	References
Transcription	cPINK1 nuclear localisation promoted by monoubiquitination; implicated in phosphorylation of transcriptional regulators	Overexpressed ΔM104PINK1-EGFP in HeLa, HEK293T, SHSY5Y; siRNA PINK1 knockdown in HEK293	[22,23]
Translation	cPINK1-induced phosphorylation at Ser^398^ of the translation elongation factor eEF1A1	Overexpressed tagged cPINK1 in AD293	[21]
Trafficking	Trafficking of mitochondria, including within dendrites and axons, via interaction of cPINK1 with Miro/Milton or PKA	Expression of ∆MTS-Pink1 in HEK293-FT, COS7; overexpression of PINK1 in SHSY5Y, PINK1^−/−^ primary cortical neurons; *Drosophila*	[24–26]
Aggregation	Direct K-48 ubiquitinated proteins to aggresomes via cPINK1-mediated phosphorylation of SQSTM1	Overexpressed tagged cPINK1 in AD293	[27]
Degradation	Sensor of proteasome capacity via cPINK1 accumulation; enhances autophagy during proteasome inhibition	MG132 proteasome inhibition	[23,27]
	Promotes α-syn degradation through autophagy via direct interaction with cPINK1 KD	Overexpression of truncated PINK1 variants in HEK293T	[28]

Beyond synthesis, cPINK1 also has the capacity to modify the pooling and processing of protein aggregates. Under conditions of cellular stress in which proteasomal degradation is overwhelmed, perinuclear aggresomes accumulate K63-ubiquitinated proteins before autophagic clearance takes over as a compensatory degradation mechanism [29]. cPINK1 is an ideal candidate for transduction of the proteasomal impairment signal to downstream aggresome machinery owing to its tightly regulated proteasome-dependent constitutive degradation, whereby the level of cPINK1 is strongly correlated with the degree of proteasomal inhibition [30]. Accumulated cPINK1 was found to divert K48-ubiquitinated proteins directly from proteasomal degradation to temporary storage in aggresomes without the need for the addition of K63–ubiquitin chain [27]. This activity was subsequently traced to cPINK1-mediated phosphorylation of SQSTM1 (also known as p62), a ubiquitin receptor responsible for the sequestration and targeting of polyubiquitinated proteins. Phosphorylation of SQSTM1 at Ser^28^ increased its affinity for ubiquitin chains and enhanced the formation of aggresomes, effectively targeting ubiquitinated proteins for autophagic degradation until proteasome capacity is restored. In addition to this, an interaction between cPINK1 and pathological α-synuclein was shown to result in autophagic clearance [28].

Of special interest in the context of neurodegeneration is the ability of cPINK1 to act as a neurotrophic factor. Neurotrophic proteins are those that regulate the survival, growth and morphological plasticity of, and synthesis of differentiation-specific proteins in, neurons [31]. cPINK1 fulfils this definition; it has been shown to direct developmental neuronal differentiation [32], promote adult neurogenesis [33], enhance neurite outgrowth [34], promote dendritic branching via phosphorylation of membrane-fusion cofactor NSFL1 [35] and stimulate neuronal plasticity by modulating the level of other neurotrophic factors (such as BNDF) in a kinase-dependent fashion [36]. Most of this activity appears tightly associated with the ability of cPINK1 to tune mitochondrial biogenesis via transcriptional programs [34] in concert with PINK1 activity at the mitochondrial surface, where it can regulate fission and fusion [37] and modulate axonal trafficking [24]. These dynamic processes are crucial regulators of neural stem cell self-renewal [38] and loss of cPINK1 may skew these processes to favour self-renewal of neural stem cells over the generation of differentiated progeny [39].

## fPINK1 as a driver of mitophagy

In the case where the association between fPINK1 and the OMM occurs in depolarised mitochondria, the import of the N-terminus across the IMM by TIM23 is inhibited. As a result, the subsequent cleavage of PINK1 by MPP and PARL do not occur, and fPINK1 accumulates on the OMM. fPINK1 then becomes activated following dimerisation and autophosphorylation of Serine 228 and 402 [14,40]. At this point, activated PINK1 can trigger several mechanisms that ultimately lead to degradation of the mitochondrion by the autophagy machinery.

### Parkin-dependent mitophagy

**Figure 2:**
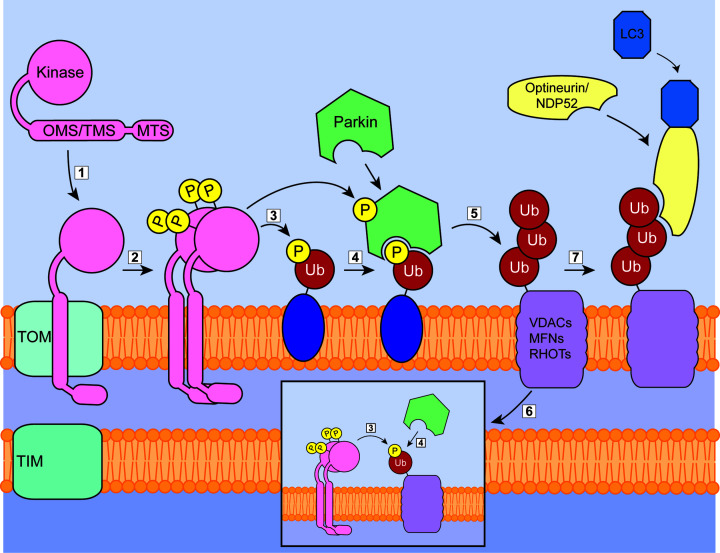
PINK1/Parkin-mediated mitophagy (1) In depolarised mitochondria, PINK1 associates with the OMM through TOM, but its import through TIM is inhibited. (2) Full-length PINK1 is then activated on the OMM through dimerisation and autophosphorylation of Serine 228 and 402. (3) PINK1 then phosphorylates several targets, including ubiquitin (Ub) that is present on various OMM proteins through the action of MARCH5. (4) Phospho-ubiquitin allows the binding of Parkin, which is also phosphorylated and activated by PINK1. (5) Activated Parkin further ubiquitinates a range of OMM proteins, which can act as (6, inset) further substrates for PINK1 and subsequent binding of Parkin, resulting in a strong positive-feedback loop. (7) Phospho-ubiquitin allows the binding of the autophagy receptors Optineurin and NDP52 and the subsequent formation of the autophagosomal membrane through the binding of LC3.

Mediating Parkin-dependent mitophagy is by far the most well-characterised role of PINK1 (Figure 2), a role that was brought to the fore by a series of landmark papers showing that PINK1 phosphorylates ubiquitin [41–45]. The action of the OMM-associated mitochondrial ubiquitin ligase (MITOL, also known as March5) results in the presence of pre-existing ubiquitinated OMM proteins [46]. These serve as the initial substrate for PINK1-mediated phosphorylation at Ser^65^ [46] which allows the recruitment of Parkin. The activation of Parkin is a multistep process: first, the N-terminal ubiquitin-like (ubl) domain of Parkin is released from its inhibitory position upon Parkin binding to phospho-ubiquitin [45,47–49]. Ser^65^ in the ubl domain can then be phosphorylated by PINK1 [50,51] which then rebinds to the unique parkin domain on the protein. This binding then results in the final activation of Parkin through the subsequent release of the catalytic RING2 domain from its autoinhibited position [52,53]. Separately to the phospho-ubiquitin recruitment of Parkin, Parkin has been proposed to be recruited to depolarised mitochondria by Miro1 [54] and mitofusin 2 [55] that have been phosphorylated by PINK1. However, recent proteomic work indicated that the role of these other phospho-proteins in the PINK1/Parkin mitophagy pathway appears to be less significant than that of phospho-ubiquitin [56]. Once recruited to the OMM and fully activated, Parkin acts to ubiquitinate numerous OMM proteins, including the voltage-dependent anion channels (VDACs), mitofusins (MFNs) and Rho GTPases (Miros/RHOTs) in both immortalised cell lines [57–59] and neuronal models [59]. An amplification cascade of Parkin-driven ubiquitination and PINK1-driven phosphorylation of ubiquitin leading to the recruitment and activation of additional Parkin results in a heavily phospho-ubiquitinated OMM. This phospho-ubiquitin is the fundamental unit that allows autophagy adaptor receptors, chiefly optineurin and nuclear dot protein-52 (NDP52) but not SQSTM1/p62 [60,61], to bind to the mitochondria and lead to degradation via the lysosome.

It has been shown in immortalised cell lines that the PINK1-mediated phosphorylation of existing ubiquitin was sufficient to induce mitophagy in the absence of Parkin [60]. Therefore, Parkin can be dispensable in the ubiquitin ligase-mediated mitophagy pathway, however the presence of the Parkin feedback cascade described above greatly up-regulates the process. Given the ability of PINK1 to drive mitophagy independently of Parkin, through this process as well as ones described below, it remains unclear as to why Parkin mutations confer such a dramatically increased risk of PD onset. One clue may be the apparent selective vulnerability of dopaminergic neurons within the substantia nigra to PINK1/Parkin deficiency. It is unclear whether the loss of either PINK1 or Parkin would have a major impact on mitophagy within these cells, as under the basal state in healthy controls PINK1 and Parkin activity in neurons is minimal [62–64]. PINK1 kinase activity is much more prevalent in primary rat astrocytes compared with cortical neurons [62], which suggests astrocytes may be more reliant upon Parkin-mediated mitophagy. This raises the possibility that the loss or dysfunction of PINK1/Parkin mitophagy in astrocytes results in neuronal death. Unfortunately, much of our understanding of PINK1 and Parkin activity stems from work completed in non-neuronal cell lines. This limits the conclusions that can be drawn regarding the importance of the proteins under pathological conditions. Further investigation into both PINK1/Parkin mitophagy and compensatory mechanisms in dopaminergic neurons, astrocytes and microglia is urgently needed.

### Parkin-independent ubiquitin-mediated mitophagy

Ongoing work is uncovering mechanisms of mitophagy that act through ubiquitin ligases other than Parkin. Of those identified so far, ariadne RBR E3 ubiquitin protein ligase 1 (ARIH1), siah E3 ubiquitin protein ligase 1 (SIAH1) and possibly glycoprotein 78 (gp78) are associated with PINK1. In each of these cases the exact mechanisms underpinning the action of the ligases is less well established than with Parkin and the prevalence of one pathway over another might be tissue or cell-type specific.

ARIH1 is an E3 ligase that appears to act in concert with cullin-RING E3 ligases [65,66] to be the main mediator of mitophagy in Parkin-downregulated cancer cells [67]. The action of ARIH1 may be controlled by PINK1 in a similar manner to Parkin, where the phosphorylation of ARIH1 can unmask the enzymatic active site and stabilise it in an active state. However, once activated and unlike Parkin, ARIH1 induces mitophagy independently of optineurin and NDP52 through an as-of-yet unidentified mechanism [67]. Unlike ARIH1, SIAH1 is down-regulated in cancer [68] and is recruited to the mitochondria through the joint action of PINK1 and synphilin 1. The normally cytoplasmic synphilin-1 was shown to directly bind PINK1 on the OMM in a manner independent of PINK1 phosphorylation [69]. The subsequent binding of SIAH1, of which synphilin is a known substrate [70], leads to the ubiquitination of OMM proteins and the binding of LC3 [69].

Another mediator of mitophagy and possible substrate of PINK1 is gp78, which is primarily localised to the ER membrane. However, mitochondria-associated ER membranes can act as a physical bridge between the ER and mitochondria (reviewed here [71]), whereby gp78 can localise to the OMM. The ubiquitination of MFN1 by gp78 in depolarised mitochondria was shown to induce mitophagy [72], and unpublished work suggests that this action is PINK1-dependent [73]. An interaction between PINK1 and gp78 was shown to control the degradation of ER PINK1 [16], but the role of PINK1 in gp78-mediated mitophagy remains to be conclusively demonstrated.

### PINK1-independent mechanisms of mitophagy

Once thought only to be the sole domain of PINK1 and Parkin, it is important to note that recent advances have uncovered multiple mitophagy receptors that are not reliant upon either protein. These receptors (including AMBRA1 [74], Bcl2L13 [75], BNIP3 [76], nix/BNIP3L [77], FKBP8 [78] and FUNDC1 [79]) are typically anchored to the OMM while presenting an LC3-interacting region (LIR) to the cytosol. The direct binding of LC3 or GABARAP to these domains can therefore induce mitophagy while bypassing the ubiquitin-dependent pathways described above. Of course, receptor-mediated mitophagy is a tightly regulated process, typically through phosphorylation; in the case of nix the phosphorylation of Ser^34^ and Ser^35^ within the LIR [80], and the formation of homodimers (abrogated by Ser^212^ phosphorylation) [81] regulate its ability to recruit LC3. In addition to these proteins, some mitochondrial lipids including cardiolipin [82] and ceramide [83] can act as mitophagy receptors.

Evidence supporting the potential for these pathways to naturally compensate lost PINK1/Parkin mitophagy is emerging. The natural up-regulation of nix-mediated mitophagy in a Parkin-deficient individual was shown to be sufficient compensation [84,85], with the individual currently not experiencing PD symptoms into their seventh decade despite carrying a mutation known to be causative of early-onset PD. It is feasible that such successful compensation of the PINK1/Parkin mitophagy pathway by nix or other receptors is underrepresented, due to the relatively low incidence rate of PINK1/Parkin mutations and their recessive inheritance pattern. Research into these alternative pathways presents a promising candidate for the development of therapeutics targeting neurodegenerative diseases featuring defective PINK1/Parkin mitophagy. However, it remains unclear how the spectrum of cytosolic cPINK1 functionality is compensated.

## fPINK1 regulates cell death via phosphorylation of apoptotic mediators

**Figure 3:**
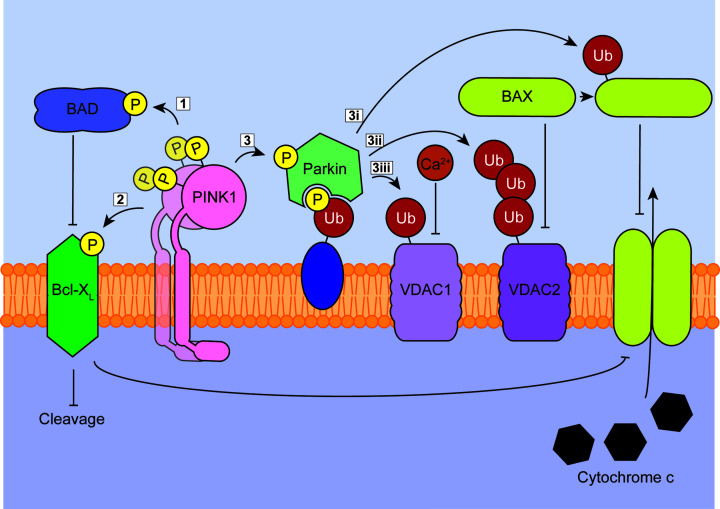
Anti-apoptotic roles of PINK1 PINK1 phosphorylation acts as an anti-apoptotic signal through several substrates. (1) Phosphorylated BAD is inhibited in its normal pro-apoptotic binding to Bcl-X_L_ on the OMM. (2) Direct phosphorylation of Bcl-X_L_ functions to prevent its cleavage, thus encouraging its inhibition of the formation of BAX pores and the subsequent release of cytochrome *c*. (3) Parkin phosphorylation also provides several anti-apoptotic influences, including: (3i) the ubiquitination of VDAC1, regulating Ca^2+^ influx; (3ii) the polyubiquitination of VDAC2, inhibiting the association of BAX and (3iii) the direct ubiquitination of BAX, inhibiting the formation of pores on the OMM.

In addition to energy production, mitochondria are a central hub for molecular events determining cell fate. Although moderate levels of mitochondrial stress and dysfunction can be tolerated by the cell given efficient mitophagy, chronic or extreme stress will eventually trigger an apoptotic response. In addition to inducing mitophagy for the removal of damaged organelles (thus mitigating stress), PINK1 is emerging as a central moderator of the apoptotic response, promoting cell survival under energetic, enzymatic and oxidative stress through broad-acting kinase activity (Figure 3; [86,87]). PINK1 has been found to phosphorylate a growing list of anti-apoptotic substrates. Some interactions of note include heat shock protein 75 (HSP75; also known as TRAP1), the phosphorylation of which protects against oxidative stress-induced cell death [88] and Bcl-X_L_, for which phosphorylation by PINK1 prevents its pro-apoptotic cleavage [89]. In addition to these pro-survival proteins, PINK1 phosphorylates BAD [87] and down-regulates apoptosis by inhibiting the binding of BAD to Bcl-X_L_/BCL-2 on the mitochondrial membrane [90].

PINK1 can also provide additional indirect anti-apoptotic pressure through the downstream action of Parkin. While complete mechanisms are yet to be resolved, recent work shows that the polyubiquitination of VDAC1 by PINK1-activated Parkin promotes mitophagy, while monoubiquitination exerts an antiapoptotic influence through the regulation of calcium influx [91]. It was also recently proposed that the ubiquitination of VDAC2 by Parkin inhibits cytochrome *c* release by inhibiting BAX association [59,92], while the direct ubiquitination of BAX also inhibits translocation to the mitochondria [93,94]. Furthermore, the ubiquitination of BAK was shown to prevent the release of cytochrome *c* by inhibiting apoptotic pore formation [92]. Together, these interactions provide potential mechanisms to underpin observations that PINK1 promotes cell survival against various pro-apoptotic stimuli in multiple cell types, including cancer [95] and neuronal models [96–98].

## Summary

- PINK1 is a broad-acting kinase that regulates biochemical pathways key to dysfunction in neurodegenerative disease.
- The primary roles of PINK1 in protein homoeostasis, mitophagy and apoptosis are highly dependent on processing and subcellular context.
- Understanding the intricacies of these roles and cellular redundancies are crucial to developing therapeutics for neurodegenerative disease.

## Abbreviations

AD: Alzhiemer's disease
ARIH1: ariadne RBR E3 ubiquitin protein ligase 1
cPINK1: cleaved PINK1
ER: endoplasmic reticulum
gp78: glycoprotein 78
KD: kinase domain
LIR: LC3-interacting region
MFN: mitofusin
MTS: mitochondrial targeting sequence
NDP52: nuclear dot protein-52
OMM: outer mitochondrial membrane
PARL: presenilin-associated rhomboid-like protein
PD: Parkinson's disease
PINK1: PTEN-induced kinase 1
SIAH1: siah E3 ubiquitin protein ligase 1
TOM: translocase of the outer membrane
ubl: ubiquitin-like
VDAC: voltage-dependent anion channel
